# The heterogeneous energy landscape expression of KWW relaxation

**DOI:** 10.1038/srep20506

**Published:** 2016-02-16

**Authors:** J. H. Wu, Q. Jia

**Affiliations:** 1 Peter Grünberg Research Center, Nanjing University of Posts and Telecommunications, Nanjing 210003, China; 2 Department of Management, Hohai University, Nanjing 211100, China; 3 Research Institute of Engineering and Technology, Korea University, Seoul 136-713, South Korea; 4 School of Life Sciences, Shandong University, Jinan 250100, China

## Abstract

Here we show a heterogeneous energy landscape approach to describing the Kohlrausch-Williams-Watts (KWW) relaxation function. For a homogeneous dynamic process, the distribution of free energy landscape is first proposed, revealing the significance of rugged fluctuations. In view of the heterogeneous relaxation given in two dynamic phases and the transmission coefficient in a rate process, we obtain a general characteristic relaxation time distribution equation for the KWW function in a closed, analytic form. Analyses of numerical computation show excellent accuracy, both in time and frequency domains, in the convergent performance of the heterogeneous energy landscape expression and shunning the catastrophic truncations reported in the previous work. The stretched exponential *β*, closely associated to temperature and apparent correlation with one dynamic phase, reveals a threshold value of 1/2 defining different behavior of the probability density functions. Our work may contribute, for example, to in-depth comprehension of the dynamic mechanism of glass transition, which cannot be provided by existing approaches.

The famous Kohlrausch-Williams-Watts (KWW) relaxation function or the stretched exponential relaxation function is an important observation in complex systems from the intricate behavior of liquids and glasses, the folding of proteins, to the structure and dynamics of atomic and molecular clusters, describing well the phenomena of important time-dependent dynamic processes^1^,^2^,^3^,^4^,^5^,^6^,^7^,^8^,^9^,^10^,^11^. The ubiquitous character of the KWW relaxation has shown irreversibility on the atomic, molecular or electronic scale and the dynamic nature of irreversible processes can be scrutinized in the context of the H-theorem to equilibrium, with the glassy state highlighting the limiting non-equilibrium behavior^1^. The dynamics of protein conformational changes clearly follows the KWW relaxation modes^2^ and geometric frustration can happen once lattice structure averts simultaneous minimization of local interaction energies^3^. KWW related slow dynamics and internal stress relaxation in bundled cytoskeletal network is essential for the mechanical properties of living cells^4^, in contrary to the stretched relaxation of flux-freezing breakdown in high-conductivity magnetohydrodynamic turbulence^5^. Most often, phenomena of the KWW relaxation are typical of glass forming liquids and other complex fluids and have been extensively investigated in such a context^1^,^10^,^12^.

The function is described by the equation of


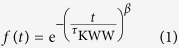


for the stretching exponential *β* between 0 and 1 (*β* = 1 is the normal exponential function) and the time *t* from 0 to  +∞ (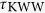 is the characteristic relaxation time)^13^,^14^,^15^,^16^,^17^.

Since there is no obvious mathematical means to analytically transform the function *f* (*t*) in spite of its simple form, so a proper resolution and understanding of the function imperatively relies on its relaxation time spectra, which is still evading due to the complexity of the function and non-closed analytic approaches used in the previous research. Nevertheless, attempts have been made to explicate the stretched exponential behaviour as a linear superposition of simple exponential decay^13^,^14^,


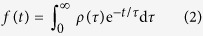


taking *τ*_KWW_ = 1 for brevity. Eq. 2 is an inhomogeneous Fredholm equation of the first kind, in which the problem is to get the function *ρ*(*τ*), provided the continuous kernel function 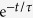 and the function *f* (*t*)^18^. *ρ*(*τ*) plays the role of the distribution of relaxation times as the probability density function of the relaxation modes. The solution of *ρ*(*τ*) can be computed from the series expansion^14^, 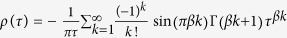. However, problems of oscillation and deviation arise due to truncations from calculating the series expansion in the non-closed form^13^,^19^,^20^. We shall use an alternative distribution, the modulus function 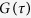
17,18, defined in the way of 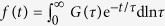. There is a simple relation between 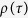 and 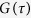, 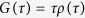. The study of the KWW relaxation is turned into the computation of 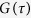,


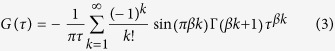


Evidently, an accurate inverse transformation of the KWW function in a closed form is of importance in applications, particularly relevant to processing experimental data^13^,^14^,^19^,^20^, but it needs tremendous efforts. From the viewpoint of the dynamic free energy distributions and heterogeneity of relaxation as well as the characteristics of a rate process, here we present a heterogeneous energy landscape scheme to obtain the relaxation time distributions of the dynamic modes of a dynamic process which is dependent on the stretching exponential. In this way, we put the stretched exponential function on a solid physical basis, resolving the dilemma that in spite of the widespread success in describing relaxation data, the function is by and large viewed as an expedient phenomenological approach short of fundamental significance.

## Results and Discussion

The concept of energy landscapes has been well explored in separate disciplines^8^,^10^. The spectrum of the KWW relaxation times implies a distribution of the free energies associating with the corresponding relaxation modes. In order to get such a distribution for a homogeneous process, we consider a global free energy random variable, 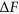 in the reduced form of the free energy 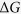 relative to the thermal energy 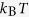 or 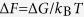 (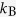 the Boltzmann constant and *T* the temperature), of a system as the sum over infinite many energy random variables (fluctuations) around its mean value from a constant random energy variable, 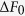, at different levels of stochastic cascading with exponential distributions^21^,^22^. Suppose 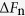 (n = 1, 2, 3, …, m, m→+∞) are those independently, nonidentically distributed random variables, with the exponential distribution of 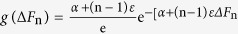, which is defined over the domain of [
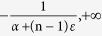
] and zero elsewhere. The two parameters have the properties of 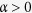 and 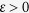. Obviously, 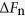 has the expectation of 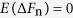 and a standard deviation 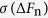 of 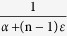. With the zero mean value and the limited magnitude of the standard deviation, 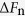 represents a fluctuating contribution to the global energy quantity of the system. The roughness strength of 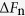 may be quantified through its standard deviation. As n increases, the measure of 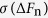 shows a harmonic-like dwindle.

We are interested in the limiting probability density distribution of the global energy variable 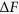 defined as 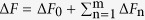 as m→+∞. In the equation, the global energy variable 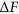 has the same expectation, set to 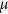, as that of the constant random energy variable 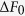 since 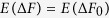. Moreover, 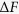 has a finite standard deviation square of 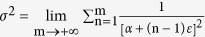
or 
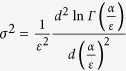
. For the sum of 
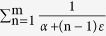
 is divergent when m→+∞, so 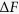 spreads over the domain (−∞, +∞). By some mathematical manipulation^23^,^24^, we are able to formulate the general probability density distribution function of the global free energy quantity 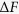 as


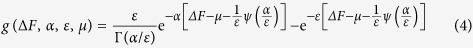


where 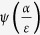 is the digamma function. 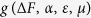 is verified as the probability density function of the global free energy distribution for the homogeneous process with the three parameters 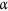, 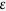, and 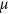.

In reality, relaxation is a rate process and the characteristic relaxation time is related to the corresponding free energy by the Arrhenius equation of 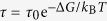 or 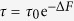 (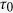 is constant)^25^,^26^. As a result, the general probability density distribution function of the global free energy quantity in Eq. 4 is converted to the relaxation time spectrum by the expression of 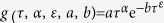, with 
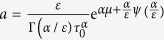
, and 
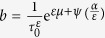
.

Furthermore, the realization of relaxation may pass a transient state during the rate process, which can go forward to a relaxed state or move back to the initial state without relaxing^25^,^26^,^27^. The rates of the forward and backward relaxation are probably correlated to the duration of dwelling on the transient state, which can be characterized by the corresponding relaxation time. Hence, the forward and backward transmission rate is assumed to have the form of 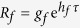 and 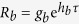 (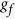, 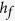, 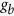, 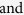 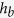 are constants), respectively. The transmission coefficient 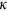 is then defined by the expression of 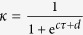, with the new constants of *c* and *d*. In consequence, the relaxation time spectrum for the homogenous process is given by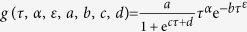.

We turn to consider the fact that the heterogeneous dynamics in glasses and other complex systems is attributed to the transitory coexistence of two dynamical sub-processes (phases) characterized by a fast and a slow relaxation rate in general^10^,^28^. In this scenario, the two sub-processes or dynamic phases contribute to the total relaxation, probably separating during relaxation and mixing afterwards. Therefore, the modulus function 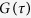 is composed of such two heterogeneous dynamic phases,


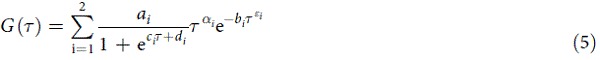


where 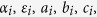 and 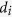 are constants for the dynamic phases *i* (*i *= 1 and 2), under the conditions that the parameters 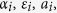 and 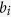
are positively valued but no such constraints on 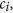 and 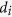. For 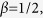 there exists an exact solution of the modulus function 
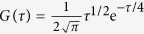
, implicitly validating the expression of 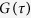
14.

**Figure 1:**
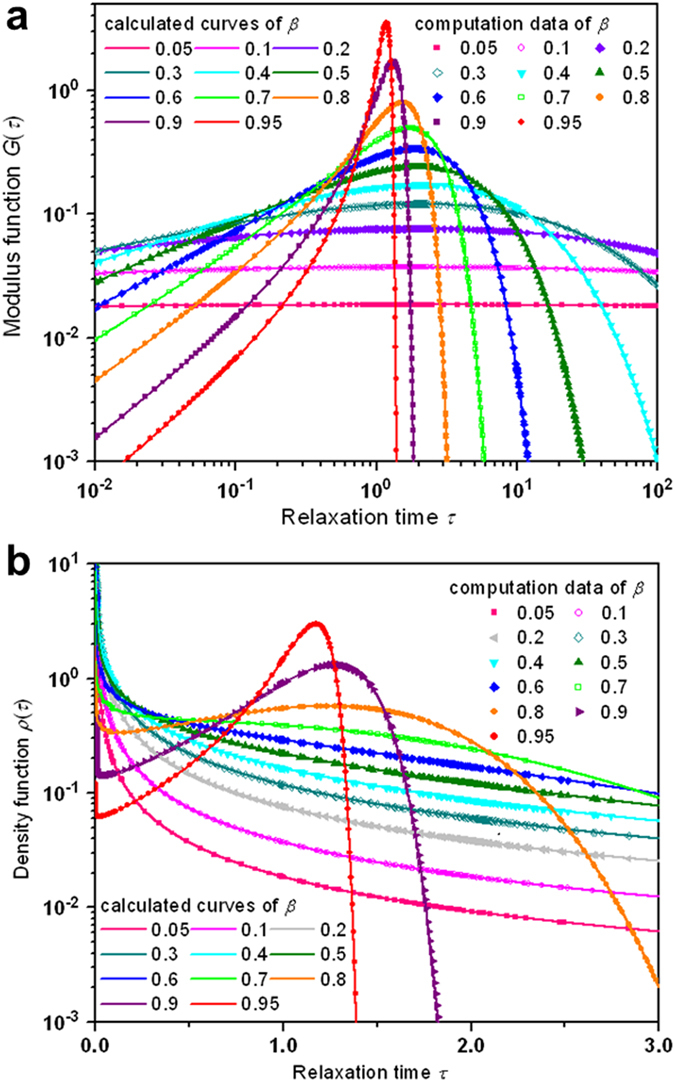
Analyses of the computational results of the KWW relaxation time spectra for values of the stretching parameter *β* between 0.05 and 0.95. The computational data points from Eq. 3 are shown in symbols and the calculated results from Eq. 5 are given in continuous curves. a, Log-log plots of the modulus function 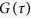 for 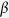 values between 0.05 and 0.95. The results manifest a strong dependence on 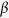, and for the same 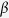, 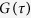 monotonically increases to attain a peak value and then decreases. b, Semi-log plots of the probability density function 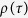 for 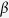 values between 0.05 and 0.95. The outcomes reveal quite different, strong dependence on 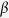, which divides the 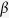 values in two ranges split by 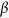 = 1/2. For the same 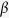 below 1/2, 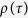 shows the behavior of monotonic decrease, in contrary to the observation that for the same 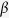 above 1/2, 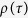 gives a rapid initial decrease, then increases to attain a peak value and then decreases.

For a given 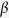, search is attempted (refer to Methods), based on the numerical data from Eq. 3 compared to Eq. 5, to find an optimized set of the parameters in the parameter space, as summarized in Table 1 for 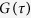, with the correlation coefficient reported to be 1. Fig. 1 shows the outcomes from the analyses of the numerical computation for 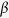 between 0.05 and 0.95. The modulus function 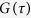 in Fig. 1a gives a strong dependence on the stretched parameter 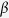. For the same 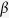, the function reveals the monotonic trend of initial increasing, attaining the maximum and then decline. Moreover, a tighter distribution is found for a larger value of 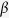 than 1/2 versus the more spread distribution of 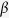 less than 1/2. The evaluation proves the accurate consensus between the numerical calculation by Eq. 3 and the derived results based on Eq. 5. In the supplemental Figure S1 we show the validity of 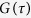 over a broader range of relaxation time. The analyses substantiate the proposition of dynamic phase coexistence in the KWW relaxation course^10^. Reviewing the parameters obtained, the transmission coefficient 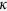 has a different weight for different values of 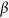, less independence on the relaxation time 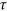 for small 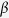 but bigger reliance large 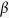, hinting a threshold value of 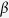 = 1/2.

The probability density distribution 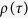 is more revealing in the exposition of the heterogeneous dynamic behavior of the KWW relaxation. The computation results are summarized in and Table 2 for 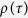, with the correlation coefficient recorded to be 1 (refer to Methods for the detailed computation procedure). Furthermore, the integration of 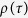 over 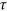 is automatically normalized, confirming the property of the probability density function and unambiguously demonstrating the self-consistence and effectiveness of our approach including the accuracy of the numerical calculation and the validity of the equations derived. Fig. 1b shows the probability density function 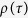 dependent on the stretched exponential 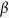. The dissimilar heterogeneous behavior is evidently manifested with a larger 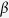 than 1/2 which shows a phenomenon of an initial decrease followed by an increase and then drop off after reaching the maximum, in contrast to the monotonic decline of the distributions with a smaller 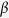 than 1/2.

**Figure 2:**
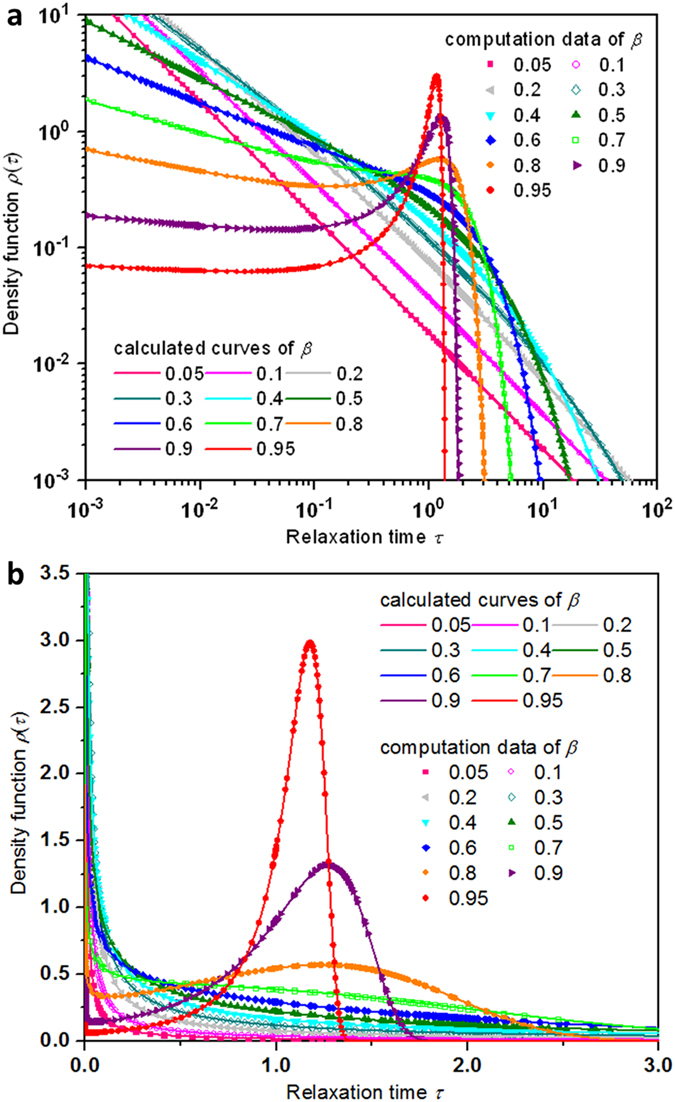
Plotting of the probability density function as a function of the relaxation time for values of the stretching parameter β between 0.05 and 0.95. (**a**) Log-log plots of the probability density function 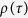 for 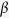 values between 0.05 and 0.95. The outcomes reveal quite different, strong dependence on 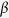, which divides the 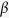 values in two ranges split by 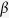 = 1/2. For the same 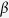 below 1/2, 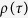 shows the behavior of monotonic decrease, in contrary to the observation that for the same 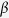 above 1/2, 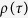 gives a rapid initial decrease, then increases to attain a peak value and then decreases. (**b**) Linear plots of the probability density function 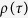 for 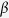 values between 0.05 and 0.95. For a small *β*, the two dynamic phases mix, but when *β* approaches 1, the major phase exclusively dominates with the minor phase disappearing, revealing the limiting behavior of 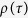 as 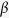 approaches 1.

The behavior of the probability density function 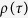 as a function of the stretched exponential becomes more distinctive if we plot the data in the log-log scale, as shown in Fig. 2a. In the figure, the numerical data calculated from Eq. 3 and the derived results based on Eq. 5 coincide over a broader range of relaxation time, showing the rationality of the approach adopted in this work in a closed, analytic form of the relaxation time spectra of the KWW relaxation. As already manifested in Fig. 1, 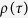 or 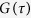 becomes more and more peaked around 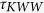 when 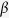 approaches 1. This limiting behavior turns out to more distinguishing by re-plotting the data in the normal coordinates, as demonstrated in Fig. 2b for the probability density function 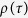.

**Figure 3:**
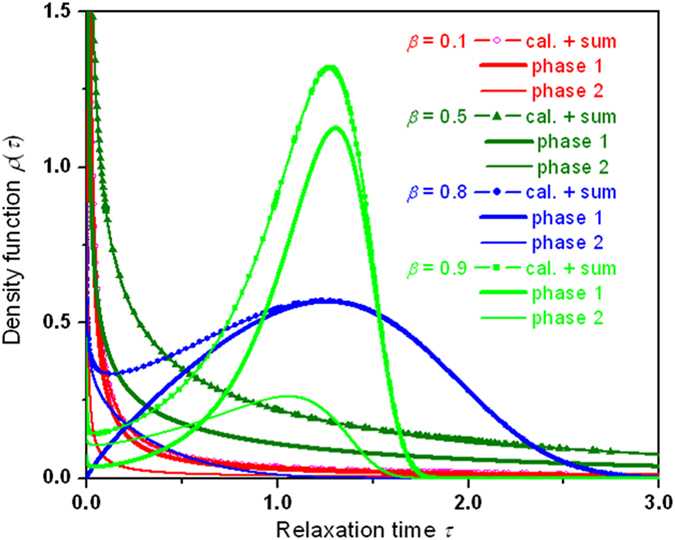
Decomposition analyses of heterogeneity of the probability density function 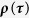 for representative values of the stretching parameter β between 0.1 and 0.9. The probability density function 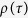 shows strong dependence on the stretched exponential 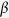 and consists of two component distributions corresponding to two different dynamic phases. The activities of the dynamic phases are quite dissimilar for 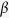
> 1/2 and 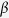
< 1/2. The analyses are performed for the same 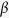 in the same color: The signs of the symbol-lines represent the computational data points from the equation of 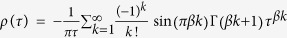 (in symbol) and the calculated results based on Eq. 5 in the continuous curve, with the thick curve for the dynamic phase 1 and the thin one for the dynamic phase 2. Data-points and curves: 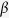 = 0.1 in red circles, 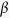 = 0.5 in olive triangles, 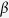 = 0.8 in blue diamonds, and 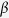 = 0.9 in green discs.

Fig. 3 gives the decomposed dynamic phases of the probability density distribution 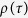 for several representative 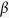 values. The value of 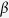 = 1/2 has a defining property, of which the two dynamic phases merge to have the same behavior. On the basis of analyzing the parameters as acquired in Table 2 and the features of the curves, the function 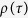 switches from the scenario that the probability densities of the two dynamic phases share analogous monotonic decrease with the relaxation time for 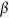 below 1/2 to the observation that the two dynamic phases present a more complicated pattern for 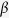 above 1/2.

**Figure 4:**
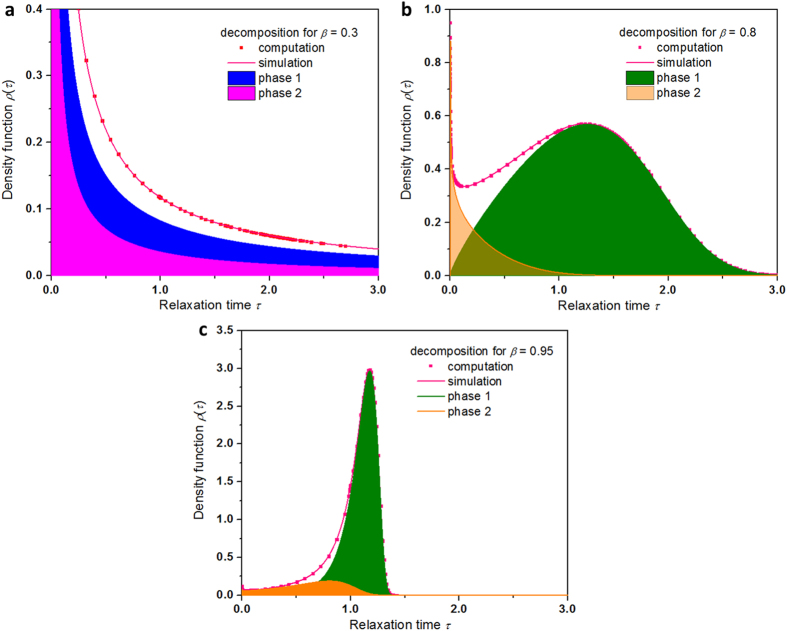
Decomposition analyses of heterogeneity of the probability density function 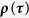 for representative values of the stretching parameter β between 0.1 and 0.95. The probability density function 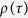 shows strong dependence on the stretched exponential 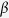 and consists of two component distributions corresponding to two different dynamic phases. The activities of the dynamic phases are quite dissimilar for 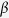
> 1/2 and 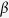
< 1/2. The analyses are performed for the same 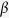. The signs of the symbol-lines represent the computational data points from the equation of 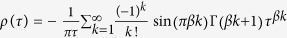 (in symbol) and the calculated results based on Eq. 5 in the shadowed regions. The limiting behavior is revealed from the peaking when 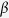 approaches 1. a, 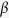 = 0.3. b, 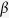 = 0.8. c, 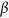 = 0.95. The bimodal feature is rapidly diminishing as *β* approaches 1, with the fast growing magnitude of the major phase against the quick weakening contribution of the minor phase, unveiling the limiting behavior of 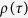 as 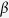 approaches 1.

In Fig. 4, we provide detailed decomposition analyses of heterogeneity of the probability density function 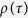 for 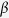 = 0.3, 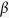 = 0.8 and 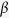 = 0.95. In general, two dynamic phases mix for small 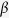, but for large 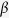 the major phase dominates while the minor phases diminishes. The limiting feature is evidently manifested in the variation of the curves from 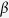 = 0.3 to 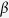 = 0.95, that is, the bimodal feature is rapidly diminishing as *β* approaches 1, with the fast growing magnitude of the major phase against the quick weakening contribution of the minor phase.

**Figure 5:**
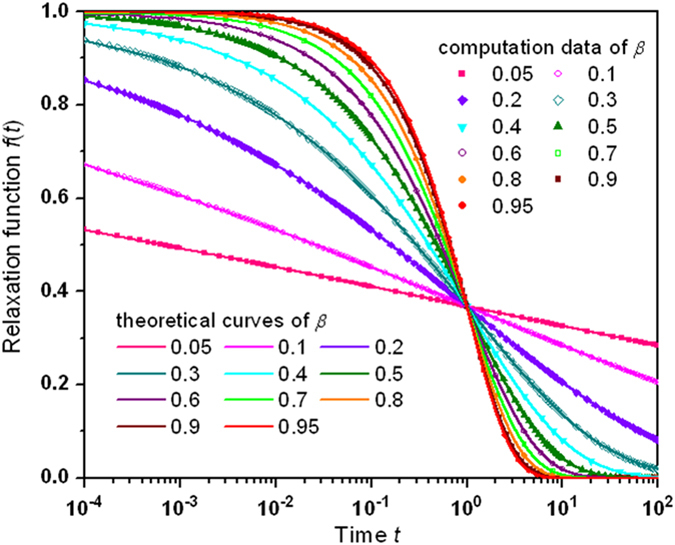
Comparison of the calculated relaxation outcome with the theoretical prediction of the KWW relaxation function 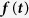 for representative values of the stretching parameter β between 0.05 and 0.95. The reverse computation data (in symbols) accurately agree with the theoretical outcomes (in curves). Labeling of the same data set of the same 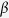 value: 0.05 (pink), 0.1 (magneta), 0.2 (violet), 0.3 (dark cyan), 0.4 (cyan), 0.5 (olive), 0.6 (purple), 0.7 (green), 0.8 (orange), 0.9 (wine), and 0.95 (red).

Fig. 5 reports the verification of accuracy in the reverse computation outcomes of the KWW relaxation function 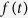 applying the formulation of 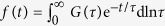 or 
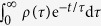
, using the parameters from Fig. 1 for 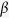 between 0.05 and 0.95 versus the theoretical curves (refer to Methods). The precise performance of the assessment is clearly exposed in the consistency of the computed data with the analytic results.

**Figure 6:**
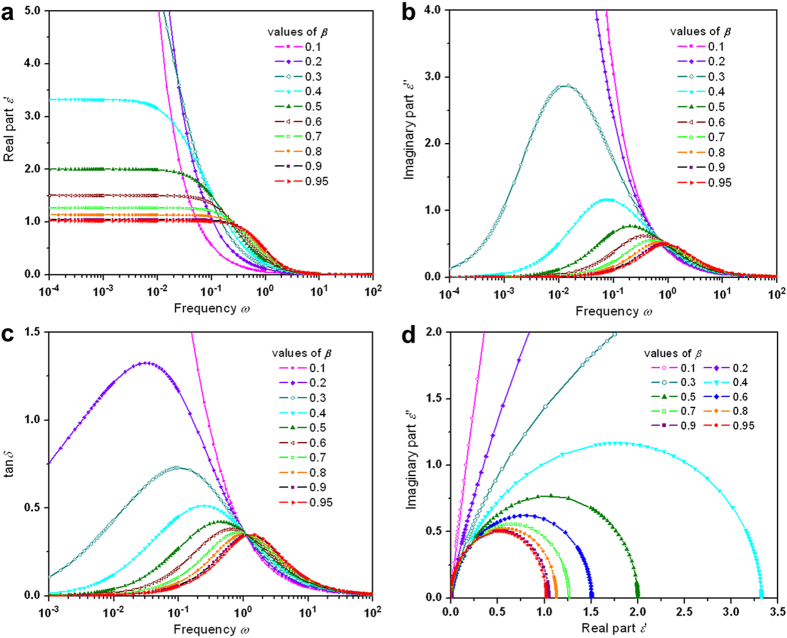
Fourier transform of the calculated KWW relaxation function 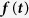 for values of the stretching parameter β between 0.1 and 0.95. (**a**) Semi-log plots of the real susceptibility 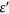 over the frequency domain. 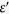 shows strong dependence on 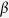, larger initial amplitude plateaus from smaller 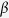. (**b**) Semi-log plots of the imaginary susceptibility 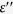 over the frequency domain. 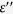 shows strong dependence on 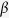, larger dissipation loss amplitudes from smaller 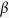. (**c**) Semi-log plots of the dissipation loss 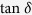 as a function of frequency. The peak position of 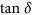 shifts right with increasing 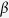, but the dissipation strength decreases. (**d**) Cole-Cole plots of the real susceptibility 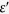 versus the imaginary susceptibility 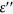 for 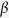 values between 0.05 and 0.95. The semi-circle-like expands outward with a decreasing value of 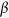. Note: Some data are out of the graphs for smaller 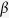 due to the plotting range for lucidity.

In order to analyze the KWW function in the domain of frequency, a Fourier transform is needed to explain dynamic susceptibilities and scattering experiments from the perspective of linear response theory^13^,^14^,^19^,^20^. Absent of analytical expression for the transform, nevertheless, previous numerical methods suffer from problems originating from approximations and truncations which yield undesired oscillations^13^,^14^,^19^,^20^. Our approach shuns the cutoff effects and scrubs out oscillations. The results of the Fourier transformation using the derived parameters from Fig. 1 are presented in Fig. 6 (refer to Methods). The susceptibilities, real part 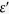 (Fig. 6a) and imaginary part 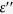 (Fig. 6b) as well as the loss tangents 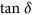 (Fig. 6c) demonstrate the relevant properties of well-defined smoothness with respect to the frequency domain and strong dependence on the stretching parameter 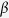. The Cole-Cole plots in Fig. 6d illustrate the susceptible relation of the relaxation, indicating a robust 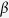 dependence.

Associated with the KWW relaxation is one important issue in condensed matter physics concerning glass transition of glass-forming materials, sharing the characteristics of free energy landscape, non-equilibrium, and heterogeneity^10^,^17^,^29^,^30^. The severe slow-down toward the glass transition temperature is linked to the decreasing 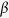 value, corresponding to the wide-spread relaxation time distribution (Fig. 1). The relation between 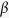 and temperature is interesting and has been examined by numerical simulations or experiments^31^, but a direct connection is still elusive. Indeed, we have tried to follow the direction to work out such a correlation between β and temperature but it requires more efforts to reach a conclusive result. Nonetheless, it may be constructive to point out that bimodal or bimodal like distributions are observed, for example, in treating the dynamic order-disorder transition in atomistic models of structural glass formers^32^. The coexistence of the bimodal order parameter distributions is clearly related to the ordered and disordered phases. In our work, the bimodal like shape is observed in the density distribution of the relaxation time. A correlation could exist between the two, but it is recognizable that more work is of necessity to establish such a direct association between the dynamic order-disorder transition and the KWW relaxation.

## Methods

This work reports a closed, analytic expression, Eq. 5, for describing the relaxation time probability density distribution function which is numerically calculated according to Eq. 3. As described below, the parameters in Eq. 5 are derived from the fit to the numerical data of Eq. 3. In this work, the fact that the two data sets coincide proves our approach, namely, Eq. 5 can excellently describe the relaxation time probability density distribution of the KWW relaxation. No other equations like Eq. 5 have been reported yet. In other words, to our knowledge, no equation other than Eq. 5 has been reported up to now to satisfactorily describe the numerical data from Eq. 3. We have performed the calculation with the help of the Mathematica and Origin software packages.

Based on Eq. 3 or the expression of


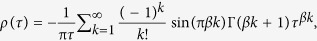


the numerical data of the modulus function 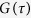 or the probability density function 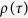 of the relaxation time distributions of the KWW relation were obtained via Mathematica for a fixed 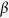 value as a function of the relaxation time. Specifically, each data point of 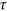 was computed up to 10^6^ terms.

Then, we used Eq. 5 or the expression of


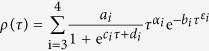


(where 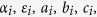 and 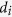 are constants for the dynamic phases 1 and 2), via the Origin program to conduct nonlinear regression of the data from the above numerical computation for a given 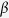. Search was repeated until an optimized set of the parameters in the parameter space was found, with the correlation coefficient reported to be 1. The results are summarized in Table 1 for 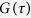 and Table 2 for 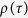.

Subsequently, the integration of 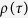 over 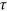 was calculated, using the parameters recorded in Table 2. It is found that 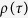 is normalized for all 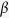 values discussed in this work.

The reverse computation of the KWW relaxation function 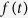 applied the formulation of 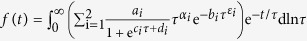 or 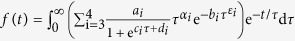. The parameters recorded in Tables 1 and 2 were used. To guarantee the accuracy of the computation, the integration was divided into segmental domains, say, [10^−8^, 10^−7^],…, [10^3^, 10^4^], and then summed up.

The Fourier transform was performed according to the expressions 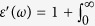

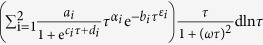
 for the real susceptibility part and 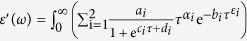

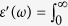

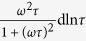
 for the imaginary susceptibility part, or 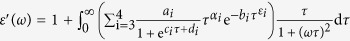 for the real susceptibility part and 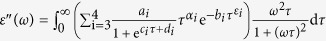 for the imaginary susceptibility part, respectively. The parameters used in the expressions were recorded in Tables 1 and 2. To secure the precision of the computation, the integration was divided into segmental domains, say, [10^−8^, 10^−7^],…, [10^3^, 10^4^], and then summed up.

## Conclusions

We have shown a heterogeneous energy landscape approach to describing the Kohlrausch-Williams-Watts (KWW) relaxation function in a closed, analytic form, which is effective both in time and frequency domains. The equations obtained ascribe the heterogeneous dynamics of the KWW relaxation to the transitory coexistence of two dynamic phases as well as the characteristics of a rate process. The relaxation time probability density distribution acquired in this way changes upon varying the stretched exponential and, in particular, it is found that *β* = 1/2 marks a crossover from a small *β* regime to a large *β* regime. Our work significantly advances the mechanism of the KWW relaxation which cannot be provided by existing schemes and offers physical insights into the dynamic processes of glass transition and other complex phenomena.

## Additional Information

**How to cite this article**: Wu, J. H. and Jia, Q. The heterogeneous energy landscape expression of KWW relaxation. *Sci. Rep.* **6**, 20506; doi: 10.1038/srep20506 (2016).

## Supplementary Material

Supplementary Information

## Figures and Tables

**Table 1:** Derived parameters of the function G(τ).

β	a_1_	α_1_	b_1_	ε_1_	c_1_	d_1_	a_2_	α_2_	b_2_	ε_2_	c_2_	d_2_
0.05	0.09694	0.05	1.10387	0.05028	0	0	0.01349	0.10229	1.05334	0.05008	0	0
0.1	0.18681	0.09996	1.17828	0.10281	0	0	0.04987	0.21331	1.10258	0.10074	0	0
0.2	0.33774	0.19926	1.19601	0.22829	0	0	0.16483	0.49152	1.21457	0.20609	0	0
0.3	0.54552	0.29706	0.99587	0.28223	0.03954	1.51629	0.1755	0.30231	0.49313	0.41286	−0.0369	−1.1461
0.4	0.72599	0.39667	0.37684	0.6248	0.01294	0.90472	0.19037	0.41549	1.01676	0.33299	0.02505	0.73563
0.5	0.14105	0.5	0.25	1	0	0	0.14105	0.5	0.25	1	0	0
0.6	0.37566	0.5999	0.17446	1.54857	0.35527	−0.9415	0.14323	1.19636	0.06925	1.68446	0.36855	−0.999
0.7	0.57003	0.70038	1.37293	0.50175	1.45002	0.35285	0.63084	1.24618	0.06321	2.58004	0.62518	−1.1133
0.8	0.18253	0.8022	1.98138	1.20762	3.79886	−3.5633	0.62092	1.81685	0.03528	4.48449	1.83817	−3.9468
0.9	1.07662	0.93241	0.08628	8.00733	−1.4811	2.50965	8.8024	0.8329	0.03668	9.05358	−3.6567	6.15082
0.95	0.78903	0.94749	0.84632	7.50918	−2.2397	2.76605	43.6189	0.8594	0.01708	19.2492	−7.2066	10.6458

Note: 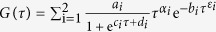.

**Table 2:** Derived parameters of the function ρ (τ).

β	a_3_	α_3_	b_3_	ε_3_	c_3_	d_3_	a_4_	α_4_	b_4_	ε_4_	c_4_	d_4_
0.05	0.09694	−0.95000	1.10387	0.05028	0	0	0.01349	−0.89771	1.05334	0.05008	0	0
0.1	0.18681	−0.90004	1.17828	0.10281	0	0	0.04987	−0.78669	1.10258	0.10074	0	0
0.2	0.33774	−0.80074	1.19601	0.22829	0	0	0.16483	−0.50848	1.21457	0.20609	0	0
0.3	0.54552	−0.70294	0.99587	0.28223	0.03954	1.51629	0.1755	−0.69769	0.49313	0.41286	−0.0369	−1.1461
0.4	0.72599	−0.60333	0.37684	0.6248	0.01294	0.90472	0.19037	−0.58451	1.01676	0.33299	0.02505	0.73563
0.5	0.14105	−0.5	0.25	1	0	0	0.14105	−0.5	0.25	1	0	0
0.6	0.37566	−0.40010	0.17446	1.54857	0.35527	−0.9415	0.14323	0.19636	0.06925	1.68446	0.36855	−0.999
0.7	0.57003	−0.29962	1.37293	0.50175	1.45002	0.35285	0.63084	0.24618	0.06321	2.58004	0.62518	−1.1133
0.8	0.18253	−0.19780	1.98138	1.20762	3.79886	−3.5633	0.62092	0.81685	0.03528	4.48449	1.83817	−3.9468
0.9	1.07662	−0.06759	0.08628	8.00733	−1.4811	2.50965	8.8024	−0.16710	0.03668	9.05358	−3.6567	6.15082
0.95	0.78903	−0.05251	0.84632	7.50918	−2.2397	2.76605	43.6189	−0.14060	0.01708	19.2492	−7.2066	10.6458

Note: 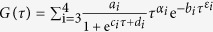.
