# Erratum: The heterogeneous energy landscape expression of KWW relaxation

**DOI:** 10.1038/srep24688

**Published:** 2016-05-20

**Authors:** J. H. Wu, Q. Jia

Scientific Reports 6: Article number: 20506; 10.1038/srep20506 published online: 02162016; updated: 05202016.

This Article contains typographical errors.

Equation 3,
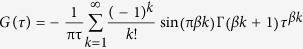


should read:
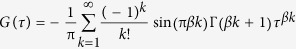


In the legend of Table 2,

“Derived parameters of the function ρ (τ). Note: 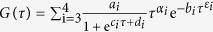.”

should read:

“Derived parameters of the function ρ (τ). Note: 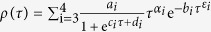.”

In the Methods section,


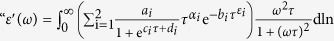
 *τ* for the imaginary susceptibility part”

should read:


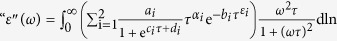
 *τ* for the imaginary susceptibility part”

In the Acknowledgements section,

“Jiangsu Provincial Natural Science Foundation of China (No. 1063-515024111)”

should read:

“Jiangsu Provincial Natural Science Foundation of China (No. BK20150823)”

