# Supplementary material for: The heterogeneous energy landscape expression of KWW relaxation
Source: Sci Rep. 2016 Feb 16;6:20506. doi: 10.1038/srep20506 (PMC4754662; doi:10.1038/srep20506)
Supplement: Supplementary Information [file srep20506-s1.doc]

**Supplementary Information**

**The heterogeneous energy landscape expression of KWW relaxation**

J. H. Wu & Q. Jia


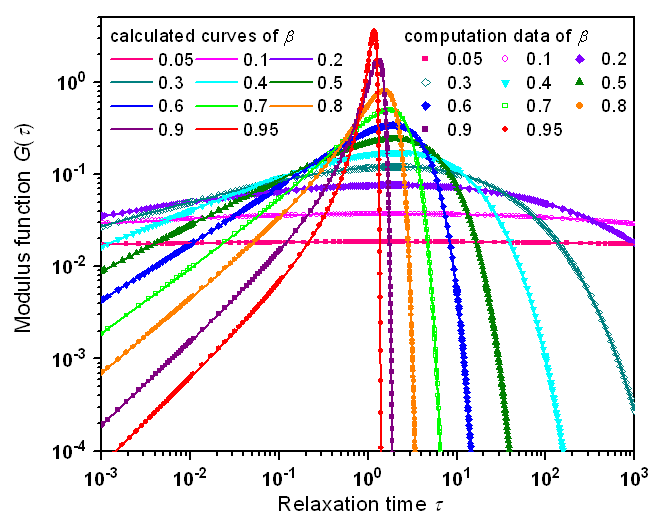


**Figure S1**  **Analyses of the computational results of the KWW relaxation time spectra for values of the stretching parameter *β* between 0.05 and 0.95 over a broad range of relaxation time.** The computational data points from Eq. 3 are shown in symbols and the calculated results from Eq. 5 are given in continuous curves. Log-log plots of the modulus function *G*(*τ*) for *β* values between 0.05 and 0.95. The results manifest a strong dependence on *β*, and for the same *β*, *Γ*(τ) monotonically increases to attain a peak value and then decreases.
